# Comparison of Direct Intersection and Sonogram Methods for Acoustic Indoor Localization of Persons

**DOI:** 10.3390/s21134465

**Published:** 2021-06-29

**Authors:** Dominik Jan Schott, Addythia Saphala, Georg Fischer, Wenxin Xiong, Andrea Gabbrielli, Joan Bordoy, Fabian Höflinger, Kai Fischer, Christian Schindelhauer, Stefan Johann Rupitsch

**Affiliations:** 1 Department of Microsystems Engineering, University of Freiburg, 79110 Freiburg, Germany; and.gabbrielli@gmail.com (A.G.); fabian.hoeflinger@imtek.uni-freiburg.de (F.H.); stefan.rupitsch@imtek.uni-freiburg.de (S.J.R.); 2 Department of Medical Informatics, Biometry and Epidemiology, Friedrich-Alexander-Universität (FAU), 91052 Erlangen, Germany; addythia.saphala@fau.de; 3 Fraunhofer Institute for Highspeed Dynamics, Ernst-Mach-Institute (EMI), 79588 Efringen-Kirchen, Germany; georg.fischer@emi.fraunhofer.de (G.F.); kai.fischer@emi.fraunhofer.de (K.F.); 4 Department of Computer Science, University of Freiburg, 79110 Freiburg, Germany; xiongw@informatik.uni-freiburg.de (W.X.); bordoy@informatik.uni-freiburg.de (J.B.); schindel@informatik.uni-freiburg.de (C.S.)

**Keywords:** presence detection, passive localization, room impulse response, acoustic localization, indoor localization

## Abstract

We discuss two methods to detect the presence and location of a person in an acoustically small-scale room and compare the performances for a simulated person in distances between 1 and 2 m. The first method is Direct Intersection, which determines a coordinate point based on the intersection of spheroids defined by observed distances of high-intensity reverberations. The second method, Sonogram analysis, overlays all channels’ room impulse responses to generate an intensity map for the observed environment. We demonstrate that the former method has lower computational complexity that almost halves the execution time in the best observed case, but about 7 times slower in the worst case compared to the Sonogram method while using 2.4 times less memory. Both approaches yield similar mean absolute localization errors between 0.3 and 0.9 m. The Direct Intersection method performs more precise in the best case, while the Sonogram method performs more robustly.

## 1. Introduction

Acoustic localization systems can provide, partly due to the comparably slower wave propagation, a high accuracy indoors similar to radio-based solutions, which are not covered by ubiquitous satellite signals of Global Navigation Satellite Systems (GNSS) [1,2,3]. For some applications, it may not be desirable to equip persons or objects with additional hardware as trackers due to inconvenience and privacy reasons. Previously, we reported coarsely about indoor localization by Direct Intersection in [4]. In this work, we report in detail on two algorithms for this application and their performances. The proposed system is categorized as a passive localization system [5] and is implemented solely with commercial off-the-shelf (COTS) hardware components.

Echolocation, such as the method used by bats to locate their prey, is a phenomenon where the reflected sound waves are used to determine the location of objects or surfaces that reflect the sound waves due to a change in acoustic impedance. This concept has been extensively used for various investigations in the physics and engineering fields, such as sound navigation and ranging (Sonar) [6,7] and even using only a single transducer for transmission and reception [8].

We draw the approach from bats, which can perceive the incoming reflected wave’s direction due to its precise awareness of head angle, body motion, and timing. While the exhaustive echolocation method of bats is not completely understood, one of the more obvious aspects is the back-scattered signals’ difference of arrival in time between left and right ears, which can be used to calculate the incoming sound wave’s direction [9]. This approach differs from approaches that more generally detect changes in the systems response of a medium, where the responses act like fingerprints. However, in application, insignificant changes in a room may lead to distortions in the response. This makes a better knowledge of the specific room necessary. In contrast, determining times-of-arrival of back-scattered waves is less dependent on the complete impulse response; we therefore chose this approach. We investigate two different algorithms based on the time difference of arrival of the first-order reflection to interpret the returned signals in a small office room of approximately 3m×4m×3m similar to [10], which are characteristic for the strong multipath fading effects that partially overlap and interfere with the line-of-sight reverberations [11]. The signal frequency employed in our experiment is significantly higher than the Schroeder frequency; therefore, we can assume the sound wave behaves much like rays of light [12]. The physiological structure and the shape of the binaural hearing conformation of bats, together with the natural and instinctive ability to perform head movements to eliminate ambiguities, enhances the echolocation and therefore guarantees excellent objects spatial localization [13]. Our system setup is a fixed structure, and we compensate the adaptive bats head movements by adding two additional microphones to the system. Furthermore, we raise the question of the performance of two approaches and compare the memory consumption and execution time.

The detection of more than one person or object is not investigated in this work.

## 2. Related Work

Indoor presence detection may be achieved through a variety of different technologies and techniques. For one, radio-frequency (RF)-based approaches have been implemented. In general, these may be classified into two different employed techniques: received signal strength indicator (RSSI)- and radio detection and ranging (Radar)-based approaches. The former offers low-complexity systems with cheap hardware [14,15], whereas with the latter one, higher accuracy may be achieved [16]. The other main concept employed in indoor presence detection is using ultrasonic waves, which are applied in active trackers indoors [17,18] and even underwater [19,20]. An entirely passive approach, as in [21], generally analyzes audible frequencies, which can include speech and potentially violate privacy regulations, similar to vision-based approaches. Acoustic solutions, which operate close to or in the audible range, can be perceived by persons and animals alike, which may cause irritation and in the worst case harm [22]. Therefore, special care has to be invested in designing acoustic location systems. While radio-based solutions are less critical in this concern, due to the fact that most organisms lack sensitivity to radio frequency signals, the frequency allocation is much more restrictive due to licensing and regulations. While LIDAR systems are highly accurate, but comparably costly, other light-based systems have gathered interest again, due to their high accuracy potential, with low systems costs and power consumption [23].

### 2.1. RF-RSSI

Mrazovac et al. [24] track the RSSI between stationary ZigBee communication nodes, detecting changes to infer a presence from it. In the context of home automation, this work is used to switch on and off home appliances. Seshadri et al. [15], Kosba et al. [14], Gunasagaran et al. [25], and Retscher and Leb [26] analyze different signal strength features for usability of detection and identification using standard Wi-Fi hardware. Kaltiokallio and Bocca [27] reduce the power consumption of the detection system by distributed RSSI processing.

This technique was then improved by Yigitler et al. [28], who built a radio tomographic map of the indoor area. The difference from the previously sampled map of RSSI values is the notification of a presence or occupancy. This general concept is known in the field of indoor localization as fingerprinting. Hillyard et al. [29] utilize these concepts to detect border crossings.

### 2.2. RF-Radar

Suijker et al. [30] present a 24 GHz FMCW (Frequency-Modulated Continuous-Wave) Radar system to detect indoor presence and to be used for intelligent LED lighting systems. An interferometry approach is implemented by Wang et al. [16] for precise human tracking in an indoor environment. Another promising approach in the RF domain is, instead of using a time-reversal approach (as Radar does), deriving properties of the medium (and contained, noncooperative objects) by means of wave front shaping as proposed by del Hougne et al. [31,32]. This approach would also in principle be conceivable in the acoustic wave domain.

### 2.3. Ultrasonic Presence Detection and Localization

A direct approach to provide room-level tracking is presented by Hnat et al. [33]. Ultrasonic range finders are mounted above doorways to track people passing beneath. More precise localization can be achieved by using ultrasonic arrays as proposed by Caicedo and Pandharipande [9,34]. The arrays’ signals can be used to obtain the range and direction-of-arrival (DoA) estimates. The system is used for energy-efficient lighting systems. Pandharipande and Caicedo [7] enhanced this approach to track users by probing and calculating the position via the time difference of arrival (TDoA). Prior to that, Nishida et al. [35] proposed a system consisting of 18 ultrasonic transmitters and 32 receivers, embedded in the ceiling of a room with the aim to track elderly people and prevent them from experiencing accidents. A time-of-flight (ToF) approach was proposed by Bordoy et al. [36], who used a static co-located speaker-microphone pair to estimate human body and wall reflections. Ultrasonic range sensing my be combined with infrared technology, as has been done by Mokhtari et al. [37], to increase the energy efficiency. In lower frequency regimes, the resonance modes of a room start to dominate the measured signals. This fact may be used to deduce source locations as proposed by Nowakowski et al. [38] (cf. [39,40]).

### 2.4. Ultrasonic Indoor Mapping

Indoor mapping and indoor presence detection are two views of the same problem. In both instances, one tries to estimate the range and direction for a geometrical interpretation. Ribeiro et al. [41] employ a microphone array co-located to a loudspeaker to record the room impulse response (RIR). The multiple reflections can be estimated from this RIR with the use of $l_{1}$-regularization and least-squares (LS) minimization, and a room geometry can be inferred, achieving a range resolution of about 1 m. A random and sparse array of receivers is proposed by Steckel et al. [42] for an indoor Sonar system. In addition to that, the authors use wideband emission techniques to derive accurate three-dimensional (3D) location estimates. This system is then enhanced with an emitter array to improve the signal-to-noise-ratio (SNR) [43]. Another approach, implementing a binaural Sonar sensor, is proposed by Rajai et al. [44]. A sensor was used to detect the wall within a working distance of one meter. In a recent work by Zhou et al. [45], it is shown that a single smartphone with the help of a gyroscope and an accelerometer can be used to derive indoor maps by acoustic probing. Bordoy et al. [46] use an implicit mapping to enhance the performance of acoustic indoor localization by estimating walls and defining virtual receivers as a result of the signals’ reflections.

### 2.5. Algorithms

The first set of methods, which are broadly applied are triangulation algorithms as described by Kundu [47]. In this work we focus on two Maximum-Likelihood approaches, similar to the one proposed by Liu et al. [48]. The first one, Direct Intersection (DI), uses a Look-up-Table (LUT) and spheres inferred from the sensors delay measurements with error margin [49], while the other one, the Sonogram method, populates a 3D intensity map with probabilities to find likely positions of the asset. Since the approaches of the two methods are different, it is likely to expect different outcomes in accuracy, precision, computational complexity, and memory requirements.

## 3. System Overview

**Figure 1:**
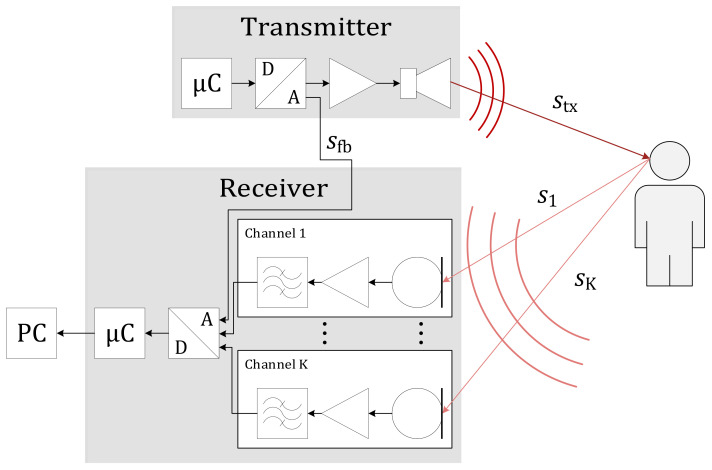
Schematic representation of the system.

The system consists of a single acoustic transmitter, a multi-channel receiver, a power distribution board, and a central computer to analyze the recorded signals. Four microphones are placed equidistantly around the speaker and connected to the receiver board. The set-up is shown in Figure 1 as it was used for the experiment reported below.

### 3.1. Signal Waveform

Due to their auto-correlation properties and the ability to maximize the Signal-to-Noise-Ratio (SNR) without increasing acoustic amplitude, swept-frequency cosine, i.e., frequency modulated chirp signals, perfectly fit our case-study [50]. Auto-correlated frequency-modulated chirps are able to provide compressed pulses at the correlator output, whose width in time space is defined as follows [51]:

(1) $$\begin{array}{c} P_{w}=\frac{2}{B}. \end{array}$$

The frequency-modulated signal employed in our experiments, $x_{\mathrm{Tx}}\left(t\right)$, is mathematically defined as follows:

(2) $$\begin{array}{cc} s_{\mathrm{tx}}\left(t\right) & =\left\{\begin{array}{cc} A\cos\left(2\pi \phi \left(t\right)\right), & \;\mathrm{for}\;0\leq t\leq T_{s}\\ 0, & \;\mathrm{otherwise} \end{array}\right.\;,\;\mathrm{with} \end{array}$$

(3) $$\begin{array}{cc} \phi \left(t\right) & =\frac{f_{\mathrm{end}}-f_{\mathrm{start}}}{2T_{s}}t^{2}+f_{\mathrm{start}}t, \end{array}$$

where *A* denotes the signal amplitude, $f_{\mathrm{start}}$ is the start frequency, $f_{\mathrm{end}}$ the end frequency, $B=f_{\mathrm{end}}-f_{\mathrm{start}}$ the frequency bandwidth, $T_{s}$ is the pulse duration, and ϕ(t) the instantaneous phase. The chirp instantaneous frequency is defined as follows:

(4) $$\begin{array}{cc} f\left(t\right)=f_{\mathrm{start}}+\frac{f_{\mathrm{end}}-f_{\mathrm{start}}}{T_{s}}t, & \;0\leq t\leq T_{s}. \end{array}$$

Taking into account the hardware characteristics of our setup, we selected a linear up-chirp pulse with amplitude A=1, $T_{s}=5\;\mathrm{ms}$, $f_{\mathrm{start}}=16\;\mathrm{kHz}$, and $f_{\mathrm{end}}=\;22\;\mathrm{kHz}$, which result in a time-bandwidth product of TB=30. The frequency response of a chirp signal directly depends on the Time-Bandwidth (TB) product. For chirps with TB≥100, the pulse frequency response is almost rectangular [52]. However, due to the hardware limitation of our setup, which do not allow a high (TB) product, the frequency response will be characterized by ripples. In order to mitigate the spectrum disturbances, we consider a window in the time domain the transmitted chirp pulse with a raised cosine window [52]. The frequency band, chirp length, and shaping window were chosen to minimize the system affecting persons and animals in hearing range. We implemented chirps, due to their property of spreading the signals energy over time compared to a single pulse to limit the maximal amplitude and resulting harmonics. While young and highly audio-sensitive people can in principle hear these frequencies, the short signal length of 5 ms compared to the repetition interval of 1000 ms further reduces the occupation of the low ultrasonic channel. Generally speaking, higher amplitudes and lower frequencies potentially increase the operation range of the system, but this comes at a health risk for humans and animals, which we seek to avoid.

### 3.2. Hardware Overview

To obtain 3D coordinates with static arrangement, a four-element microphone array is sampled, as well as a feedback signal. This array records the incoming echo wave with different time of arrival, depending on the incoming signal direction. Since unsuitable hardware can affect the system’s performance [53], both the microphones and speaker were tested for correct signal generation and reception in an anechoic box.

### 3.3. Data Acquisition

Each microphone’s signal was preconditioned before the digitization by the multi-channel analog-to-digital converter, which was chosen to provide each channel with the identical sample-and-hold trigger flank before conversion. Each frame consists of the signal from each microphone and a feedback, which is recorded as an additional input to estimate and mitigate playback jitter. The first layer of digital signal processing is to compress the signal, extracting the reverberated acoustic amplitude over time and removing the empty room impulse response (RIR).

#### 3.3.1. Channel Phase Synchronization

Initially, we calculate the convolution of the feedback channel signal $s_{\mathrm{fb}}$ with our known reference signal $s_{\mathrm{ref}}$ in its analytic form to obtain the RIR and retrieve the time of transmission from the compressed signal $y_{\mathrm{fb}}$, as shown in Equation (5), where j denotes the imaginary unit.

(5) $$y_{\mathrm{fb}}=\left|\left(s_{\mathrm{fb}}⊛s_{\mathrm{ref}}\right)+j\;\cdot \;H\left(s_{\mathrm{fb}}⊛s_{\mathrm{ref}}\right)\right|$$

This compressed analytic form $y_{\mathrm{fb}}$ of the feedback signal $s_{\mathrm{fb}}$ (see Figure 1) ideally holds only a single pulse from the transmitted signal, if the output stage is impedance matched. Searching for the global maximum returns both time of transmission and the output amplitude.

(6) $$a_{\mathrm{out}}=\max_{t\overset{}{\to }t_{0}}\;y_{\mathrm{fb}}\left(t\right)$$

In the following, we refer to the start time of a transmission as $t_{0}$, all other channels’ time scales are regarded relative to $t_{0}$. Therefore, the signals of the microphone channels are truncated to remove information prior to the transmission. The ring-down of small office rooms is in the order of 100 ms, so the repetition interval of consecutive transmissions is chosen accordingly to be larger. This prevents leakage of late echos into the following interval, which would result in peaks being recorded after the following interval’s line-of-sight. The remaining signal frames from all microphones are compressed with the same approach as the feedback channel, shown in Equations (5) and (6), to extract each channel’s compressed analytic signal $y_{i}$ and line-of-sight detection time $t_{i}$.

#### 3.3.2. Baseline Removal

**Figure 2:**
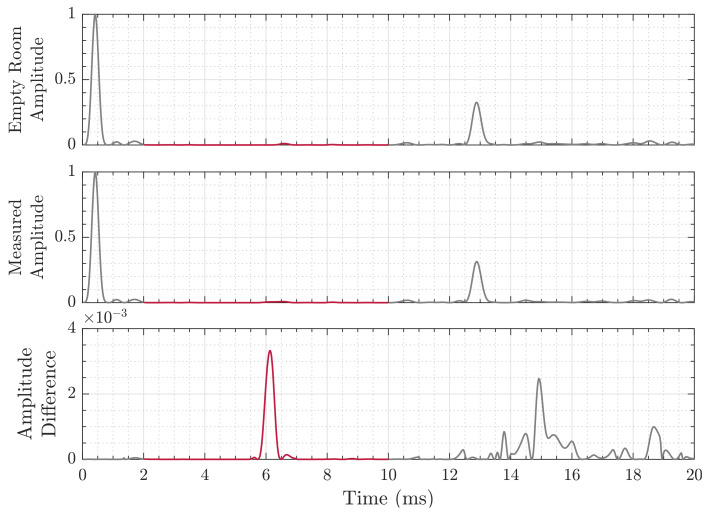
Exemplary magnitude plot of the compressed analytic signal, i.e., RIR, with (**top**) the baseline drawn from an previous recording of the empty room, (**middle**) the room with a person in it, and (**bottom**) the difference of the two above. The red highlighted line in the center marks the area of interest due to geometric constraints. Note the changed scale of the ordinate in the bottom plot.

In the following, we refer to the acoustic channel response after the line-of-sight as the echo profile. An example of such echo profiles is shown in Figure 2. While the line-of-sight signal ideally provides the fastest and strongest response, large hard surfaces, like desks, walls, and floors return high amplitudes, which are orders of magnitude above a person’s echo. For a linear and stable channel, we can reduce this interference from the environment by subtracting the empty room echo profile from each measurement, following the approach of [54]. This profile loses its validity if the temperature changes, the air is moving, or objects in the room are moved, e.g., an office chair is slightly displaced. A dynamic approach to create the empty room profile is updating an estimation, when no change is observed for an extended time or alternatively using a very low-weight exponential filter to update the room estimation. In this work, the empty office room was sounded *N* times directly before each test and averaged into an empty room echo profile ${\bar{y}}_{i}^{\circ }$ for each channel *i* as denoted in Equation (7), to assure unchanged conditions and reduce the complexity of the measurements. The removal itself is then, as mentioned above, the subtraction of the baseline from each measurement, as in Equation (8), under the assumption of coherence.

(7) $${\bar{y}}_{i}^{\circ }=\mathrm{mean}\left(y_{i}^{\circ }\right)$$

(8) $${\tilde{y}}_{i}=y_{i}-{\bar{y}}_{i}^{\circ }$$

#### 3.3.3. Time-Gating

For our approach we assume some features of the person, such as being closer to the observing system compared to the distant environment objects, like chairs, tables and monitors, while another area of reverberations is in the close lateral vicinity of the system, consisting, e.g., of lamps and the ceiling. This is exploited by introducing a time gate, which only allows for non-zeros values in the interval of interest as in Equation (9) (also compare Figure 2).

(9) $${\tilde{y}}_{\mathrm{tg},i}=\left\{\begin{array}{cc} {\tilde{y}}_{i}, & \mathrm{for}\;t_{\min}<t<t_{\max}\\ 0, & \mathrm{otherwise} \end{array}\right.$$

Another assumption is that of a small reverberation area on the person. We assume the points of observation from each microphone to be sufficiently close on a person to overlap. The latter assumption introduces an error, which limits the precision of the system in the order of 10 cm [55], which we deem sufficient for presence detection, as a person’s dimension is considerably larger in all directions. This estimation is based on the approximate size of a person’s skull and its curvature with respect to the distance to the microphones and their spacing. The closer the microphones and the further the distance between head and device, the more the reflection points will approach each other. If we regard a simplified 2D projection, where a person with a spherical head of radius $r_{H}\approx 10\;cm$ moves in the y-plane only, the position of a reflection point $R=\left(x_{R},\;z_{R}\right)$ on the head can be calculated by

(10) $$\begin{array}{cc} x_{R} & =x_{C}-r_{H}\sin\left(\alpha_{R}\right),\;\mathrm{and}\\ z_{R} & =z_{C}-r_{H}\cos\left(\alpha_{R}\right), \end{array}$$

where $x_{C}$ and $z_{C}$ are the lateral and vertical center coordinates of the head and $\alpha_{R}$ is the reflection angle. The latter is calculated through

(11) $$\alpha_{R}=\tan^{-1}\frac{x_{C}+\frac{d_{M}}{2}}{z_{C}},$$

with the distance $d_{M}$ between the microphone and sender. The origin is set as the speaker position. By geometric addition, the distance between two such reflection points can be calculated and reach the maximum value if the head moves towards the center. In this case, the reflection points would be on the opposing sides of the head and result in a mismatch of $2\;r_{h}$. The other extreme is laterally moving to a infinite distance, which increases the magnitude of $x_{C}$, while the distance between microphone and speaker stays constant; therefore, the reflection points converge to a single point of reflection. In this work, the distance between head center and speaker remained above 120 cm, with a projected error distance of about 1.3 cm.

#### 3.3.4. Echo Profile

**Figure 3:**
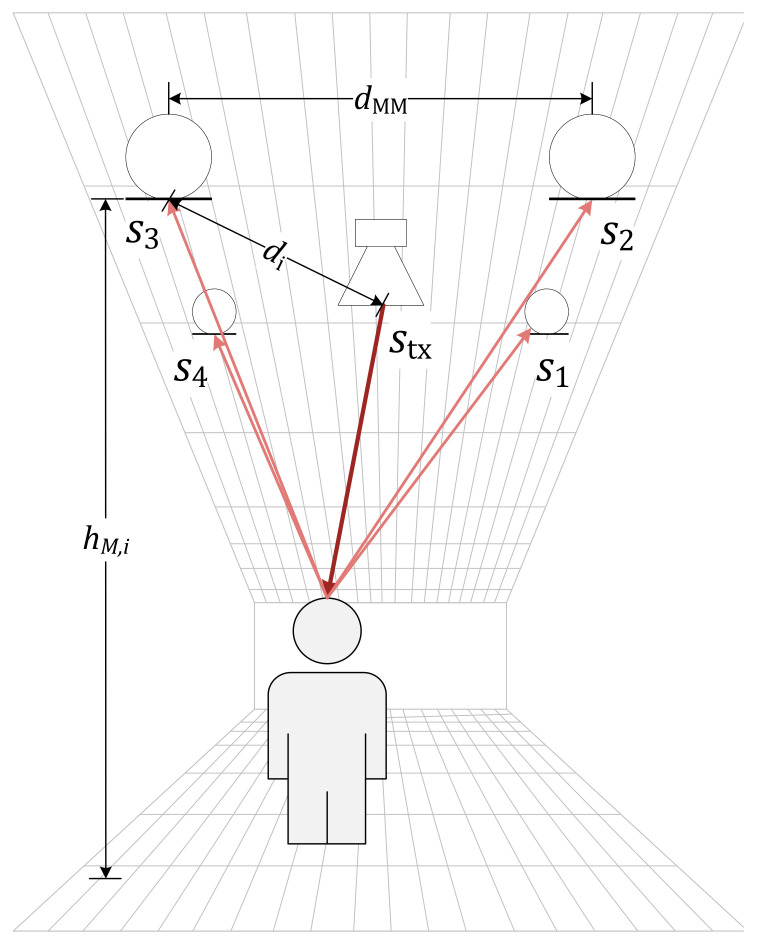
Experimental setup for K=4 receivers spaced by $d_{\mathrm{MM}}\approx 0.2\;m$. The transmitted signal $s_{\mathrm{tx}}$ is observed as reflected signals $s_{i}$ by the system located near the ceiling of the room.

During the experiment, the reflected signals from the floor, walls, tables, and chairs have a very high amplitude. This interference can lead to masking the echo from the target object. To reduce the effect of the interference, the empty room profile is used to subtract the target impulse response from the input impulse response. If we define the reflection from objects other than the target object as noise, we can increase the signal-to-noise ratio with this method. The empty room impulse response is also called empty room echo profile in this work. In Figure 2, the upper plot is the empty room impulse response, where the experiment room is cleared of most clutter. The middle plot is the room with single static object as target, shown in Figure 3. The lower plot shows the result of subtraction between the the second and first plot, and the scale is adjusted for clarity.

#### 3.3.5. Distance Maps

Look-up tables are calculated before the experiment to estimate the travel distance of a signal from the speaker to each microphone under the assumption of a direct reverberation from a point at position $\vec{x}$ in the room and linear beam-like signal propagation. This grid is formed by setting the center speaker as origin and spanning up a 3-dimensional Cartesian coordinate system of points $\vec{x}$ through the room in discrete steps. We limit the grid to the intervals $X_{1}$ to $X_{3}$ in steps of 1 cm to decrease the calculational effort and multipath content under the prior knowledge of the rooms geometry as follows:

(12) $$\begin{array}{cc} \vec{x} & =\left(x_{1},x_{2},x_{3}\right)\in X,\;\mathrm{where}\\ X & =\left\{X_{1}\times X_{2}\times X_{3}\right\}\subset R^{3}. \end{array}$$

The look-up table approach serves to minimize the processing time during execution. The distance maps provide pointers to convert from binary sampling points to distance points. Each sub-matrix contains the sum of distance between each point in the room to the corresponding *i*th microphone at the position ${\vec{x}}_{M,i}$ and to the speaker at position ${\vec{x}}_{S}$, which cover the flight path of the echoes, as in Equation (13):

(13) $$M_{i}\left(\vec{x}\right)=∥\vec{x}-{\vec{x}}_{S}∥+∥{\vec{x}}_{M,i}-\vec{x}∥.$$

Therefore, the resultant entries in matrices *M* depend on the geometric arrangement of speaker and microphones, and the matrix size corresponds to the area of detection, as in Equation (12).

### 3.4. Data Processing

#### 3.4.1. Direct Intersection

The main assumption for this approach (Algorithm 1) is that the highest signal peak in the observation window of each channel indicates the position of interest, as visualized in Figure 2. Each channels’ peak index defines the radius $r_{i}$ of a sphere around each microphone, which is contained in the point cloud $L_{i}$. While ideally those spheres overlap in exactly the point of reverberation, in practical application, where noise, interference, and jitters are present, this is not the case. To compensate this error, we pad the sphere by Δr additional points in the radius until all spheres overlap and the unity of valid estimation points $U_{L}$ is not empty. The sphere radius widening Δr can be used as an indication of each measurement’s quality, as a low error case will require little to no padding, while in high-error cases, the required padding will be large. Another approach is to use a fixed and small padding, which will ensure only measurements of high quality to be successful, but will fail for high error scenarios.
**Algorithm 1:** Direct Intersection Estimation [56,57].
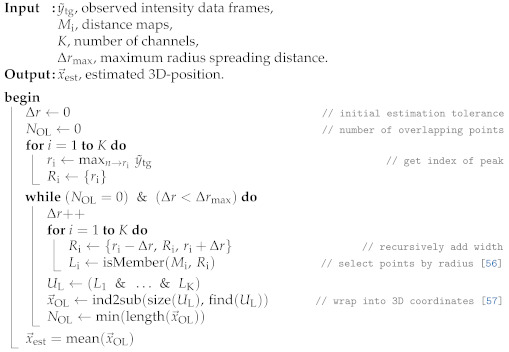


#### 3.4.2. Sonogram

The Sonogram approach (Algorithm 2) leverages available memory and processing power to build a 3D intensity map. This approach utilizes the entire echo profile difference shown in Figure 2 (bottom) and maps them into the 3D distance map explained in Section 3.3.5, with the assumption that the highest peak corresponds to the source of reverberation. The multiplication of impulse amplitude that corresponds to the same coordinates is used as an indication of possible reverberation source. Therefore, the maximum result would have the highest likelihood of being the reverberation source location.
**Algorithm 2:** Sonogram Estimation [58,59].
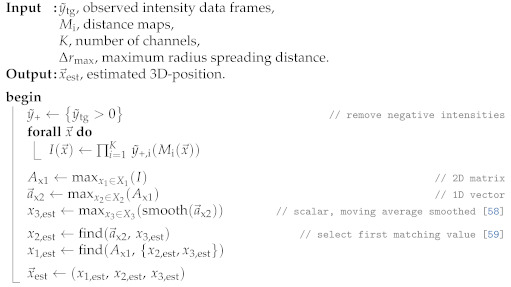


## 4. Experiments

### 4.1. Set-Up

In the experiment, we use a mock-up representing a person’s head as the experiment target. The hard and smooth surface of the object is intentional for the sake of usability and to remove unintended movements from our measurements at this early stage. In the set-up shown in Figure 3, the central speaker emits the well-known signal $s_{\mathrm{tx}}$, and the reflected echoes from the target $s_{1}$ to $s_{4}$ are recorded by the microphone array around the speaker. The depiction in Figure 3 is exaggerated for clarity.

Table 1 shows the spherical coordinates, i.e., radial distance *r*, azimuth angle θ, and elevation angle ϕ of the target inside the room, with the center of the device as the reference point. The device is positioned on the ceiling, oriented downward. For each position, we measure the distance for the assumed acoustic path with a laser distance meter Leica DISTO^TM^ D3a BT for reference. As mentioned above, the coordinate system’s point of origin is set to the center of the device, the x-axis is set perpendicular to the entrance door’s wall, and increasing towards the right, the y-axis is parallel to the line of sight from the door and increasing towards the rear end of the room, and the z-axis is zero in the plane of the device (upper ceiling lamp level) and decreasing towards the floor. The two-dimensional depictions are shown in Cartesian coordinates to provide clarity, while the detection results are done in spherical coordinates.

### 4.2. Results

#### 4.2.1. Room Properties and Impulse Response

**Figure 4:**
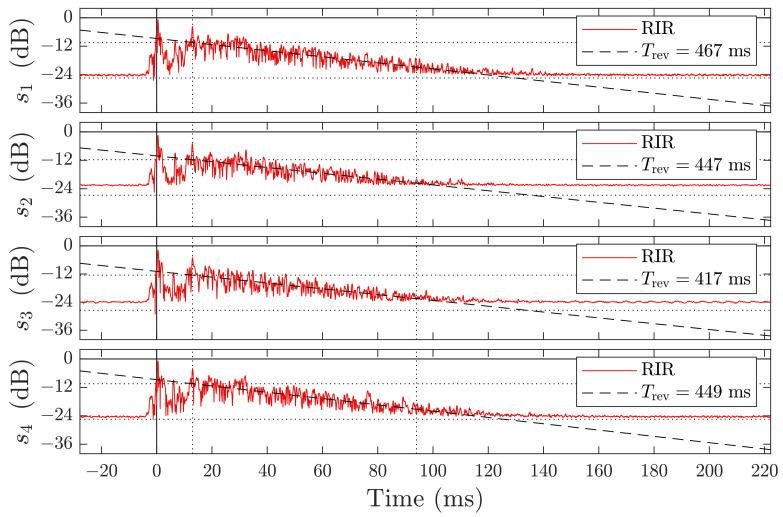
Empty room’s impulse response magnitude of a linear chirp ($T_{s}=5\;ms$, 16 to 22 kHz) in logarithmic scale for all 4 channels $s_{1}$ to $s_{4}$. The red line indicates the mean response over 100 measurements, with a linear fit indicated by a black dashed line in the interval between 13 to 94 ms (dotted vertical lines) to approximate the reverberation time constant $T_{\mathrm{rev}}$ of the room, given in the legend of each channel’s subplot. The upper horizontal dotted line indicate the fit’s level at t=13 ms, while the lower indicates an additional drop by −20 dB.

**Figure 5:**
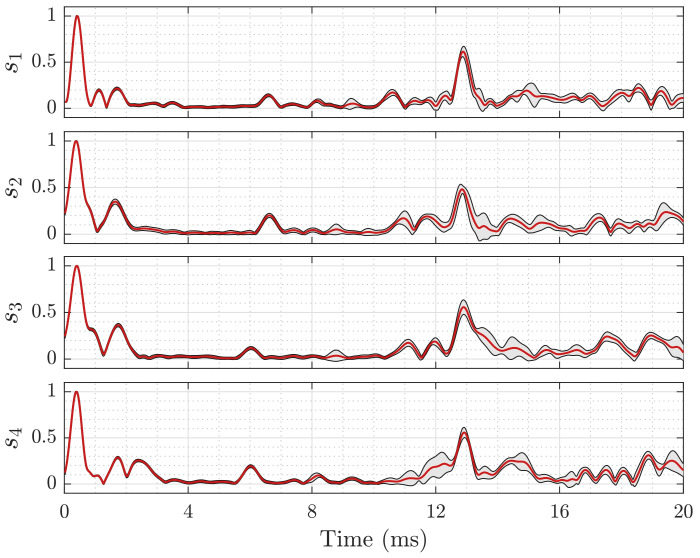
First 20 ms of the empty room’s amplitude response for all 4 channels $s_{1}$ to $s_{4}$. The red line indicates the mean response over 100 measurements, the grey envelope the ±3σ region. The first peak marks the line-of-sight arrival time and is used for time synchronization.

In preparation for the later experiments, we sounded the room 100 times as described in Section 3.3.2 to record the baseline profiles shown in Figure 4 and Figure 5. This recordings were taken one time and served as a reference for all later experiment runs. During the recordings, the room was left closed and undisturbed.

The room exhibits a different room response for each microphone, as illustrated in Figure 4. We divide the response into four parts: line-of-sight, free space transition, first order echoes, and higher order echoes, i.e., coda [60]. The signal remains in the room for more than 100 ms, before it drops below the noise floor. The definition of the reverberation time from Sabine requires a drop of the sound levels below −60 dB [61,62], for which the low signal-to-noise ratio of less than 24 dB does not suffice. Therefore, we adapted a fractional model and extrapolated the reverberation from a drop of 20 dB. The resulting mean reverberation time of the room is approximately ${\bar{T}}_{\mathrm{rev}}\approx 445\;ms$, which corresponds to a dampening factor δ≈15.5 s^−1^ and a Schroeder frequency of approximately $f_{\mathrm{sch}}\approx 230\;\mathrm{Hz}$, which is far below the transmission band. In this work, we focus on the response in the parts-free space transition and first-order echoes to estimate a person’s position. A close-up of the first three parts of the room response is shown in Figure 5.

The recordings still show significant variances in each channel at varying positions, e.g., in the uppermost subplot of Figure 5 from 15 to 16ms. Below 8 ms, these intervals with increased variances do not occur, indicating a stable channel. The signals’ interval close to zero contains strong wall and ceiling echos. Note the very strong reverberation peak at 12.5 to 13.5 ms that is caused by the floor. As our area of interest does not fall within this distance, we omit it for analysis as well. Hence, the time-gate limits as introduced in Section 3.3.3 are $t_{\min}=3\;ms$ and $t_{\max}=8\;ms$.

If we transfer the room dimensions into the wavelength space, hence

(14) $$\Lambda =\frac{l}{\lambda_{g}}=\frac{\ell \;f_{g}}{c},$$

with *c* as the speed of sound and *l* the room dimensions in the respective Cartesian direction, we can draw an estimator from [63] for the number of modes below the reference frequency $f_{g}$ as

(15) $$N_{\mathrm{mode}}=\frac{4\;\pi }{3}\left(\Lambda_{x}\;\Lambda_{y}\;\Lambda_{z}\right)+\frac{\pi }{2}\left(\Lambda_{x}\;\Lambda_{y}+\Lambda_{y}\;\Lambda_{z}+\Lambda_{z}\;\Lambda_{x}\right)+\frac{1}{2}\left(\Lambda_{x}+\Lambda_{y}+\Lambda_{z}\right).$$

This lets us calculate approximately $15\times 10^{6}$ modes below 16 kHz and $40\times 10^{6}$ modes below 22 kHz, which leaves about $25\times 10^{6}$ modes in the sounding spectrum in-between. If we regard the number of eigenfrequencies below the Schroeder frequency, Equation (15) yields $N_{\mathrm{sch}}\approx 73$ modes that strongly influence the sound characteristics of the room [64].

#### 4.2.2. Direct Intersection

**Figure 6:**
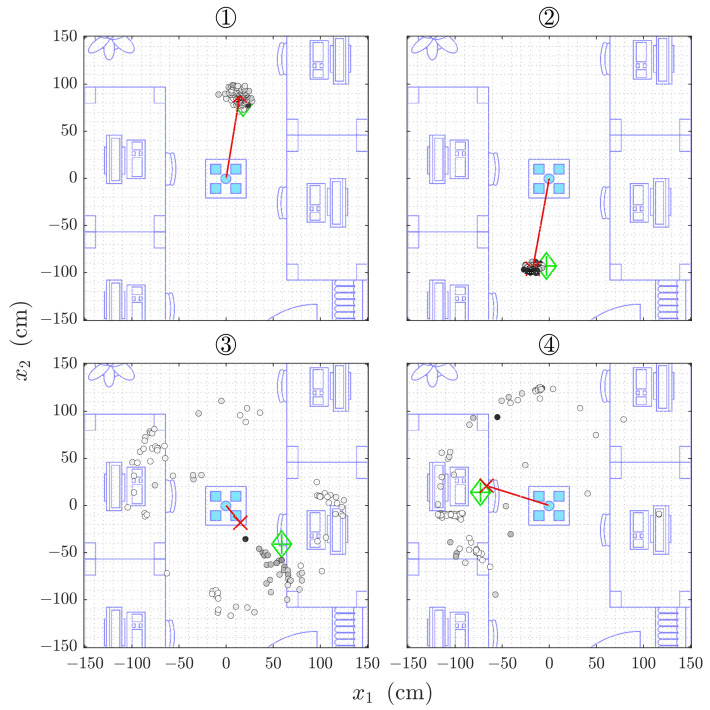
2D projection of 100 estimations of 3D positions ① to ④ by Direct Intersection. The single estimations are indicated by the black circled markers, the red cross marks the Cartesian averaged position and is highlighted by the red line to the origin, and the green diamond indicates the reference position. The points’ infill is proportional to the observed intensity relative to the radius spreading (darker is higher).

The localization by Direct Intersection from all 100 runs is shown for each of the four reference positions in Figure 6. While the statistical evaluation is performed in spherical coordinates due to the geometric construction during the estimation, this overview plots, as well as those for the Sonogram localization are drawn in Cartesian coordinates that allow for easier verification and intuitive interpretation. The lateral spread of the estimation point cloud in Figure 6 ① is misleading as the points are situated on a sphere around the origin. The projected lateral extent is almost entirely due to the angular errors.

Positions ① and ② show a distance estimation deviation of $\sigma_{r}\approx 10\;cm$, as well as azimuth and elevation angle errors of $\sigma_{\theta }\approx \sigma_{\phi }<5{}^{\circ }$ for both Direct Intersection and Sonogram localization (compare Table 2 and Table 3). For positions ③ and ④, which are situated closer to the desks, the deviation increases to almost 40 cm in distance and almost arbitrary azimuth angles with a $\sigma_{\theta }\approx 120{}^{\circ }$ and more, but a far less affected elevation angle estimation with a $\sigma_{\theta }<10{}^{\circ }$. The deviations are calculated around the mean estimator for each value. For simplicity of interpretation, the mean error for each dimension is shown in Section 4.2.3.

**Figure 7:**
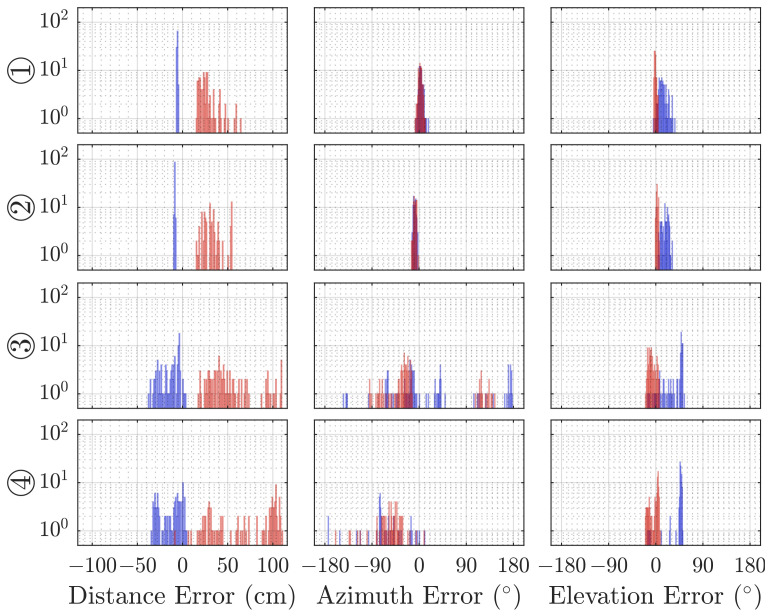
Histograms of the error in estimation compared to the reference over 100 localization repetitions at each position by DI (blue) and Sonogram (red) estimation. Each row depicts the 3 degrees of freedom for each position.

The error distributions for each dimension are shown in Figure 7, where each column depicts one of the spherical dimensions (radius, azimuth angle, and elevation angle), while each row represents the results from the reference position indicated to the left of the plot. For the first two positions, the distributions are almost unimodal, but for the latter two, this does not hold true, making the mean value and standard deviation unsuitable estimators.

**Figure 8:**
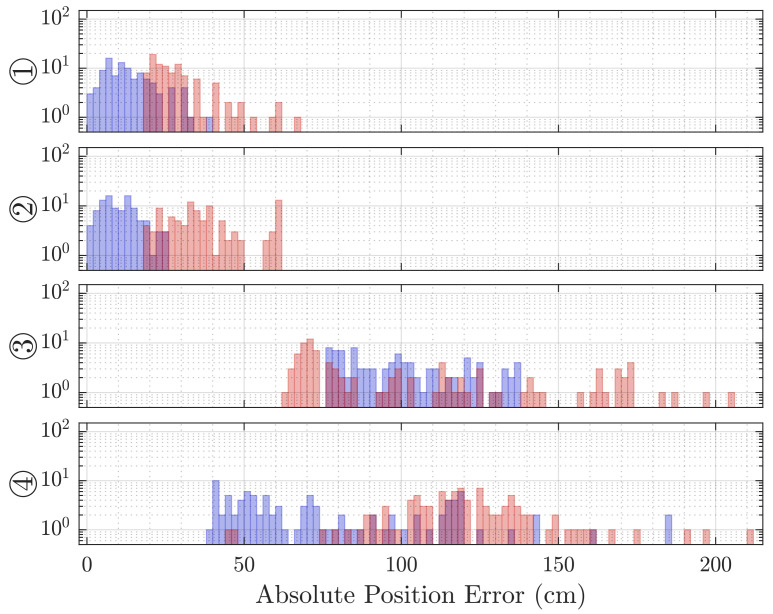
Histograms of the absolute distance error in estimation compared to the reference over 100 localization repetitions at each position by DI (blue) and Sonogram (red). Each row depicts the 3 degrees of freedom for each position.

The distribution of the error in the absolute distance between the estimated positions and reference positions (see Figure 8) is likewise a few dozen centimeters for the first two cases, but around 1 m for the latter two. If we recall the reference positions from Table 1, the true distances are between 1 and 2 m, which puts the error in the same order as the expected value.

**Figure 9:**
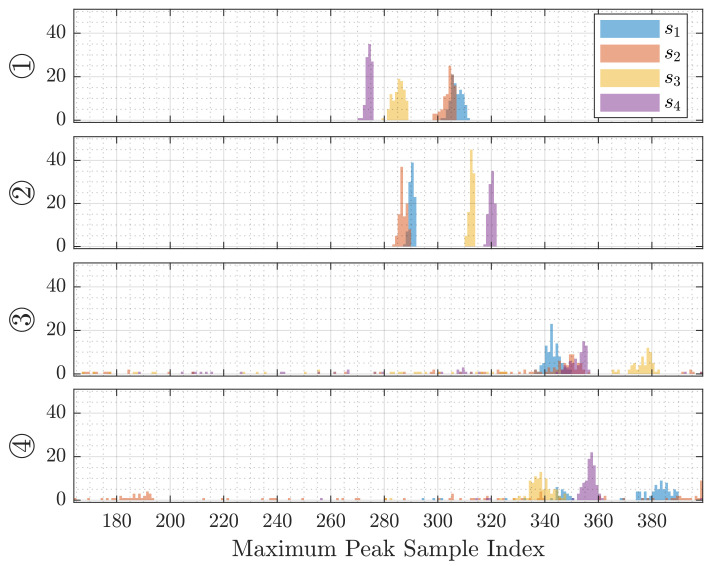
Histograms of the highest peak position of each microphone’s channel over 100 localization repetitions at each position by DI. Each row depicts the 3 degrees of freedom for each position.

The Direct Intersection method allows for an investigation into the time variance of the detected maximum peak, which is depicted in Figure 9. In the first two cases, we observe unimodal distributions of around 10 samples in width, while the latter cases show detected peaks all over the interval.

#### 4.2.3. Sonogram

**Figure 10:**
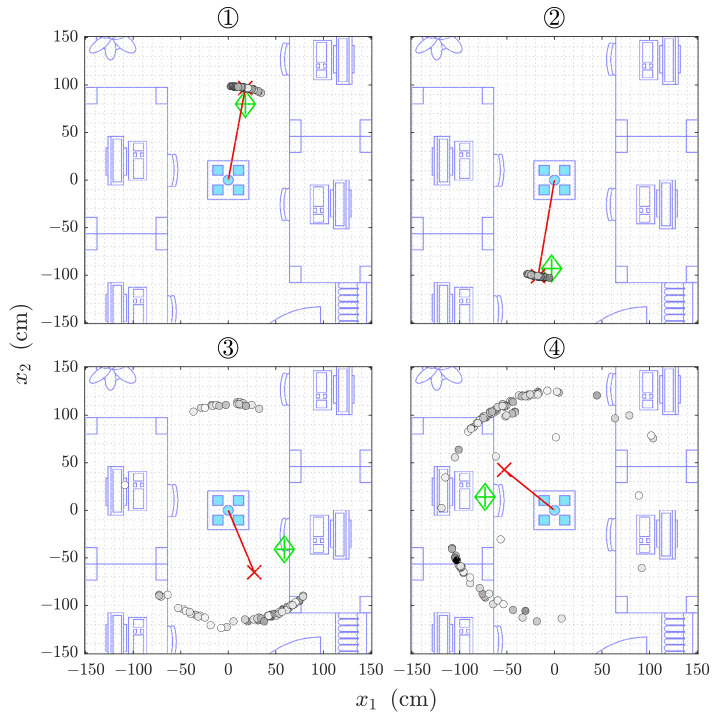
The same position estimation plot as in Figure 6 for positions ① to ④ but by Sonogram. The reference position is given by the green diamond, the averaged estimation by the red cross, and each circle represents a single estimated position. The circles’ infill is proportional to the observed intensity.

The Sonogram localization on the same data as before in Section 4.2.2 is shown in Figure 10 for all four cases. The lateral distribution of the estimated locations is not following the spherical shape as closely as is the case for those by Direct Intersection estimations (compare, e.g., Figure 6 ①).

Similar to before, the method performs well in the cases ① and ②, exhibiting small deviations (see Table 3), but far less precise with the largest deviation increase in the azimuth angle as well. The corresponding mean errors to the reference positions are listed in Table 4.

The cases ③ and ④ display two larger clusters of estimated positions, which leads to the bimodal error distributions in Figure 7.

The absolute error is similarly distributed around lower values for the former two cases and widely spread for the two latter cases (see Figure 8). Note that the error distribution plots for the Sonogram are of slightly different horizontal scale, as no errors below 20 cm were observed, while the observed maximal error exceeds 200 cm.

**Figure 11:**
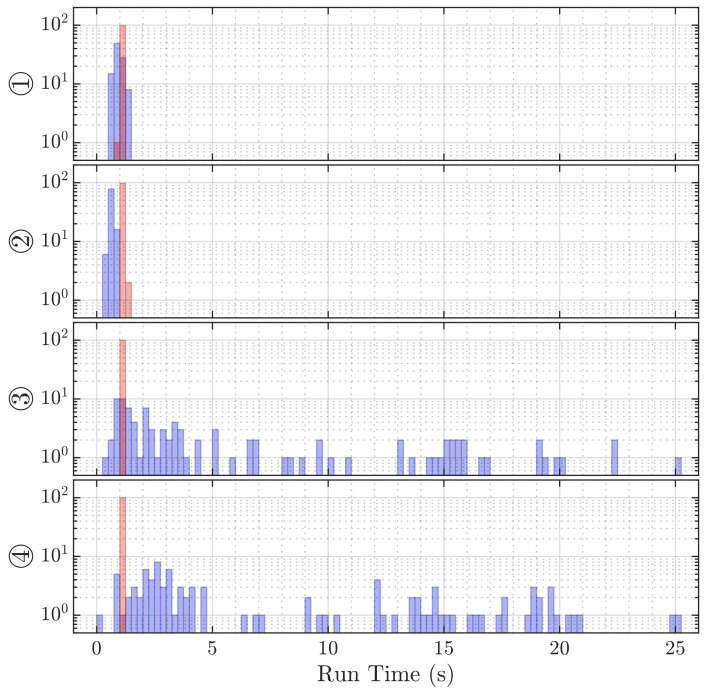
Histograms of the execution time of 100 localization repetitions at each position by DI (blue) and Sonogram (red).

**Figure 12:**
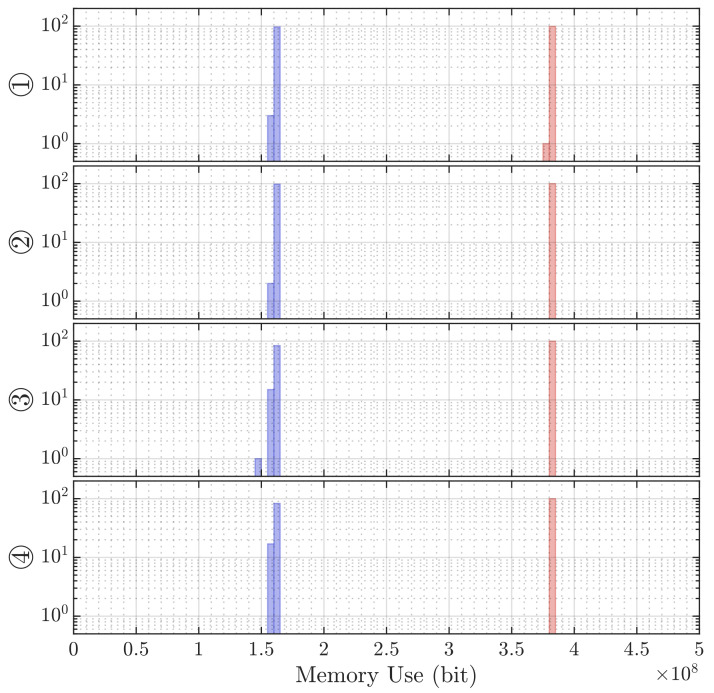
Histograms of the memory allocation during 100 localization repetitions at each position by DI (blue) and Sonogram (red).

Lastly, the performance of both algorithms with regard to execution time is listed in Table 5 and mean required memory in Table 6. The distribution of those measures is shown in Figure 11 and Figure 12. The Direct Intersection method requires roughly 2.4× less memory than the Sonogram localization. With a best-case mean execution time of 0.66 s, the former algorithm is almost 1.7× faster than the best case mean of the latter method, while the worst-case mean—almost unchanged for the Sonogram approach—is with a factor of 7.1 for the Direct Intersection by far slower than the worst case mean execution time of the Sonogram method.

The Direct Intersection execution time varies strongly, as we observe it anywhere between 0.25 and 25.0 s; thus, without further limitations, it does not allow for a well-confined prediction of the localization algorithm’s execution time.

## 5. Discussion

### 5.1. Localization

The localization methods discussed in Section 4 are based on the time of arrival of the line-of-sight reflection from the target. This is possible because the frequency-modulated signal in our experiments is significantly higher than the Schroeder frequency of the room. The Direct Intersection method provides throughout all cases distance estimations that are too short, while the Sonogram-based localization returns distance estimations that are longer than the reference (compare Figure 7). Regarding the absolute error distribution, we observe that the Direct Intersection method performs more accurately, especially in the better cases ① and ②, as well more precise in the first three of the four observed cases, as drawn from Figure 8. The possible cause of the degradation of both methods performance for cases ③ and ④ is in the peak detection algorithm, as Figure 9 shows a wide error range of detected possible peaks. While this was observed specifically for the Direct Intersection method, this also implies the low signal-to-noise ratio of the underlying echo profile, and consequently also affects the Sonogram estimation. Interestingly, the lower estimation errors for cases ① and ② implicate a better performance for the larger distances than the closer ones, which is counter-intuitive from a power perspective, but if we recall the empty room impulse responses shown in Figure 5, where noise is included as the curves’ variance, and compare it to the magnitudes of a person’s signal in Figure 2, the difference in magnitude is in the same order. For higher distances, the variance increases, as fluctuations in the speed of sound cause phase distortions, but for lower distances, interference effects dominate. The frequency band of the chirp between 16 and 22 kHz sets the wavelength range to approximately 2.2 to 1.6 cm, which is close to the distance between reflection points on a person’s head, as shown above in Section 3.3.3. Proximity to objects increases interference as well, which explains the lower performance in the closer positions ③ and ④, where the projected distance onto the sensor system’s aperture between the person and the wall, screen, and desk is reduced. If we regard the error distributions of each position in Figure 7 again, the angles and distances roughly fit non-line-of-sight paths, especially for the Sonogram method.

### 5.2. Performance

The Direct Intersection method requires less than half the memory for its computations compared to the Sonogram method, as the information is very early condensed in the peak selection part of the algorithm. The index look-up is in itself a cheap operation, but due to the sphere-spreading loop to decrease the probability of the algorithm not returning any valid position at all, comes at higher execution duration. The observed worst case for Direct Intersection is with 25 s so high that no real-time tracking is possible anymore. If we look closer at Figure 6 ③, we see that the estimation point gray scale infill is proportional to the inverse spreading factor, so darker colors mean less radial spread before intersecting points could be found. The notion that including strong outliers by allowing the sphere thickness to be spread so far is not confirmed if we consider Figure 6 ④.

## 6. Conclusions

Both methods show mean distance estimation errors ranging between approximately 0.3 and 0.9 m for objects in distances between 1.2 and 1.7 m, with angular errors between 2 ${}^{\circ }$ and 138 ${}^{\circ }$ in azimuth, 1 ${}^{\circ }$ and 7 ${}^{\circ }$ in elevation. The Sonogram Estimation allows for analysis of room response in more detail, and the results are more accurate (i.e., average error) in three out of four observed cases, but inversely, the precision (i.e., error variance) of the Direct Intersection is higher in three of the cases. The Direct Intersection method allows for less expensive computation by reducing maximum radius spreading, while the Sonogram method’s cost can be reduced effectively by limiting the vertical search interval, e.g., to the clutter free area above the desks. For a full-range sounding of the room, we observed that the locations close to the clutter area are estimated worse regarding both accuracy and precision. For a pragmatic operation on hardware with higher memory limitations the Direct Intersection method will perform faster and with similar precision and accuracy, and can be limited in execution time by restricting the sphere radius spreading at the cost of not being able to estimate the position for several intervals. We esteem further investigation into limiting the degradation of the estimation process by single unreliable channels as most promising for improving passive acoustic indoor localization.

## Figures and Tables

**Table 1:** Reference Positions.

Position	*r* (m)	θ (${}^{\circ }$)	ϕ (${}^{\circ }$)
①	1.58	77	59
②	1.70	−92	57
③	1.23	−35	54
④	1.26	169	54

**Table 2:** Direct Intersection Estimated Positions.

Position	*r* (m)	θ (${}^{\circ }$)	ϕ (${}^{\circ }$)
①	1.83 ± 0.14	81 ± 4	61 ± 1
②	2.01 ± 0.11	−100 ± 3	61 ± 1
③	1.92 ± 0.37	4 ± 96	59 ± 4
④	2.12 ± 0.25	−58 ± 135	60 ± 3

**Table 3:** Sonogram Estimated Positions.

Position	*r* (m)	θ (${}^{\circ }$)	ϕ (${}^{\circ }$)
①	1.85 ± 0.10	80 ± 4	58 ± 2
②	2.03 ± 0.11	−100 ± 3	60 ± 2
③	1.77 ± 0.26	−41 ± 69	47 ± 7
④	1.96 ± 0.34	31 ± 119	51 ± 9

**Table 4:** Mean Error for Direct Intersection and Sonogram.

	Direct Intersection			Sonogram		
Position	r (m)	θ (${}^{\circ }$)	ϕ (${}^{\circ }$)	r (m)	θ (${}^{\circ }$)	ϕ (${}^{\circ }$)
①	0.25	3	2	0.27	2	1
②	0.31	8	4	0.34	8	3
③	0.69	39	5	0.53	6	7
④	0.87	47	6	0.70	138	3

**Table 5:** Runtime Performance: Time.

	Direct Intersection	Sonogram
Position	Time (s)	Time (s)
①	0.94 ± 0.17	1.14 ± 0.07
②	0.66 ± 0.13	1.20 ± 0.02
③	6.38 ± 6.60	1.10 ± 0.01
④	8.58 ± 7.12	1.10 ± 0.01

**Table 6:** Runtime Performance: Memory.

Direct Intersection	Sonogram
$\mathrm{Memory}\;\left(\times 10^{8}\;\mathrm{bit}\right)$	$\mathrm{Memory}\;\left(\times 10^{8}\;\mathrm{bit}\right)$
1.600 ± 0.004	3.840 ± 0.002

## Data Availability

The data presented in this study are available on request from the corresponding author.

## Abbreviations

The following abbreviations are used in this manuscript:

MDPI	Multidisciplinary Digital Publishing Institute
DI	Direct Intersection
DoA	Direction of Arrival
FMCW	Frequency-Modulated Continuous-Wave
LS	Least-Squares
RF	Radio-Frequency
RIR	Room Impulse Response
RSSI	Received Signal Strength Indicator
SONO	Sonogram
SNR	Signal-to-Noise Ratio
TDoA	Time Difference of Arrival
ToF	Time of Flight
